# Correction: Kim et al. The Value of Posterior Cervical Angle as a Predictor of Vaginal Delivery: A Preliminary Study. *Diagnostics* 2021, *11*, 1977

**DOI:** 10.3390/diagnostics12071647

**Published:** 2022-07-06

**Authors:** Eun-Ju Kim, Ji-Man Heo, Ho-Yeon Kim, Ki-Hoon Ahn, Geum-Joon Cho, Soon-Cheol Hong, Min-Jeong Oh, Nak-Woo Lee, Hai-Joong Kim

**Affiliations:** Department of Obstetrics and Gynecology, Korea University School of Medicine, Seoul 02841, Korea; zzuya90@naver.com (E.-J.K.); heojiman.md@gmail.com (J.-M.H.); akh1220@korea.ac.kr (K.-H.A.); geumjoon@korea.ac.kr (G.-J.C.); novak082@korea.ac.kr (S.-C.H.); mjohmd@korea.ac.kr (M.-J.O.); haijkim@korea.ac.kr (H.-J.K.)

In the original publication [1], the Basic Science Research Program, through the National Research Foundation of Korea, NRF-2019R1I1A1A01064098, as a funder to Ho-Yeon Kim, was not included. The correct Funding information should be: This research received funding from the Basic Science Research Program, through the National Research Foundation of Korea, NRF-2019R1I1A1A01064098 as a funder to Ho-Yeon Kim. 

The authors apologize for any inconvenience caused and state that the scientific conclusions are unaffected. This correction was approved by the Academic Editor. The original publication has also been updated.
